# Association between Workplace Risk Factor Exposure and Sleep Disturbance: Analysis of the 2nd Korean Working Conditions Survey

**DOI:** 10.1186/2052-4374-25-41

**Published:** 2013-12-27

**Authors:** Yong-Seok Heo, Sei-Jin Chang, Shin-Goo Park, Jong-Han Leem, Sung-Hwan Jeon, Bum-Joon Lee, Kyung-Yong Rhee, Hwan-Cheol Kim

**Affiliations:** 1 Department of Occupational and Environmental Medicine, Inha University Hospital, Incheon, South Korea; 2 Department of Preventive Medicine, Institute of Occupational & Environmental Medicine, Yonsei University Wonju College of Medicine, Wonju, South Korea; 3 Department of Occupational and Environmental Medicine, Inha University School of Medicine, Incheon, South Korea; 4 Occupational Safety and Health Research Institute, KOSHA, Incheon, South Korea

**Keywords:** Sleep disturbance, Physical risk factor, Chemical risk factor, Biological risk factor, Psychosocial risk factor, Workplace, Korean Working Conditions Survey

## Abstract

**Objectives:**

Sleep is essential for human beings to live and work properly. This study was conducted to investigate the relationship between occupational exposures to workplace risk factors and sleep disturbance in Korean workers.

**Methods:**

The data were drawn from the second Korean Working Conditions Survey (KWCS); a total of 7,112 paid workers were analyzed. The independent variables were occupational exposures such as physical, chemical, biological, and psychosocial risk factor in the workplace, and psychosocial risk factor was divided into five categories (job demand, job control, social support, job insecurity, lack of reward). We estimated the relationship between various occupational exposures and sleep disturbance using multivariate logistic regression analysis.

**Results:**

The results showed that people who exposed to physical, chemical, biological, and psychosocial (high job demand, inadequate social support, lack of reward) risk factors were more likely to increase the risk of sleep disturbance. Furthermore, after adjusting for general and occupational characteristics, we found significant positive associations between exposures to physical (odds ratios [OR] 1.47, 95% confidence interval [CI] 1.05-2.07) and psychosocial (high job demand (OR 2.93, 95% CI 2.16-3.98), inadequate social support (OR 1.57, 95% CI 1.14-2.15), lack of reward (OR 1.45, 95% CI 1.08-1.96)) risk factors and sleep disturbance.

**Conclusion:**

These results suggest that occupational exposures to physical and psychosocial workplace risk factors are significantly related to sleep disturbance.

## Introduction

With economic development, emphasis on increasing the quality of life of individuals tends to grow, including an increasing interest in sleep quality. In spite of individual differences, sleep, which accounts for one third of a day, is essential for human life [1], for the tired and exhausted body to recover [2], to relearn information encountered during the day [3], and to maintain good mental health [4]. Therefore, it is important to sleep appropriately; a sleep impediment can cause many problems in daily life. Sleep disturbance in humans can cause physical health problems, like cardiovascular disease [5], hypertension [6], and obesity [7]; and mental health problems, like daytime sleepiness [8], fatigue, cognitive impairment [9], affective disorder [10], and absenteeism [11]. In particular, extensive research on the association between hypertension and sleep disturbance has been published [12,13]. In that literature, it is known that increasing adrenocorticotropic hormone (ACTH) and cortisol levels because of sleep disturbance or a lack of sleeping time can lead to hypertension [14] by overactivation of the autonomic nervous system. Conversely, use of hypertension drugs can cause sleep disturbance [15]. In addition, sleep disturbance can affect the process of recovering from physical health losses, like occupational injuries or accidents [16], and mental health losses, like impairment of judgment, memory, and concentration ability. This, in turn, can result in the decreased productivity of workers [17].

Sleep disturbance is defined as disorders of sleep quality, quantity, and timing. Prevalences of sleep disturbance for the past year range from 4.4% to 13.4% [18]. In Korea, 17% of the general population has sleep disturbance, and 37.3% of people who suffer from sleep disturbance complain of severe daytime physical fatigue [19]. In the research of Ohayon et al., women are usually 1.4 times more likely to have sleep disturbance than men, and with an increase in age, sleep disturbance becomes more common; half of people aged 65 or more experience sleep disturbance [20]. Moreover, it is known that sleep disturbance is more common among retired people and homemakers, and those with little formal education and low socioeconomic status. However, other studies have shown that those with more education are at greater risk of sleep disturbance, so the research on the association between sleep disturbance and educational level has not been consistent [20-22]. In addition, sleep disturbance differs by ethnicity [20,23]. Virtane et al. found that workers who work more than 55 hours a week have more difficulty falling asleep and suffer from shortened sleeping hours, compared with workers who work 35–40 hours a week [24]. Typical causes of temporary sleep disturbance are psychological stress from tests or examinations, side effects of medication, diseases like depression [18], and caffeine or daytime alcohol consumption [19]. Long working hours [24] and shift work [25-27] are representative occupational causes, and recent studies have shown that these causes interrupt natural biorhythms or cause sleep disturbance through irregular working hours. Among these causes, night shift work can reduce the quality of sleep by disrupting circadian rhythms, and it leads to fatigue and low productivity. Moreover, night shift work can reduce the quality of life by negatively affecting social relationships with family or friends [28]. Long working hours, which eliminate the recovery period for fatigue by shortening sleep duration, can reduce the psychological and physical quality of life of workers [29].

In a study of the International Labour Organization (ILO), it was suggested that the appropriate working period per day is no more than 8 hours [30]. In a study of the Organization for Economic Cooperation and Development (OECD), in Korea, the mean working time per year is 2,193 hours, in other words, above 8 hours a day [31]. Therefore, workers spend as much time sleeping as in the workplace, and during the working process, exposure to various workplace hazardous risk factors will have an effect on the worker's state of health.

There are many ways of categorizing workplace hazardous risk factors, and they can usually be classified into 3 groups, that is, physical risk factors (vibration, noise, high or low temperatures, etc.), chemical risk factors (organic solvents, mist, dust, fumes, chemical agents, etc.), biological risk factors (infectious agents, etc.) and psychosocial risk factors (stress, etc.) [32]. Many studies have been conducted in Korea on the association between occupational stress [33] or shift work [34] and sleep disturbance, but studies examining any potential association between physical, chemical, biological, and psychosocial risk factors and sleep disturbance simultaneously are rare. In the US, Japan, and Europe, concern about sleep disturbance has existed for a long time, so many studies have been conducted. Many studies have been limited to small-scale groups or specific occupations. In case of studies being targeted to wage workers, like our study, some studies have been conducted to analyze risk factors for sleep disturbance using a nation-wide representative sample [10,33]. However, studies on the association of various workplace hazardous risk factors and sleep disturbance have been uncommon, except a French cohort study [35]. Therefore, this study was conducted to search for an association between physical, chemical, biological, and psychosocial hazardous risk factors and sleep disturbance in Korean wage workers, using data from the second Korean Working Conditions Survey (KWCS) performed by the Occupational Safety and Health Research Institute in 2010. Through the study, we hope to help workers achieve a healthy sleep life.

## Materials and methods

### Study subjects

This study was based on data acquired from the second KWCS, which was conducted from May 2010 to September 2010 by the Occupational Safety and Health Research Institute. The KWCS, which is a survey about factors in the workplace environment, health, safety, and occupational stress, was first conducted in 2006. Subjects of the second survey were 15-year-old or older workers who worked at least one hour in the week prior to the interview for income. The quality of the second KWCS was assured by the high external and content validity and reliability [36]. All of the participants, 10,019 people, were directly asked questions by interviewers. Among all of the participants, in the end 7,112 wage workers were included in this study, after giving weight to 6,220 respondents under the base of the number of family members from the 2011 population census.

### Methods

#### General characteristics

General characteristics included gender (male or female), age (<30 years, 30–39 years, 40–49 years, 50–59 years, or ≥60 years), educational status (middle school or less, high school, or college or more), smoking habit (non-smoker, past smoker, or current smoker), drinking frequency (non-drinking, once a week, or more than twice a week), diagnosis of hypertension (yes or no), diagnosis of obesity (yes or no), and depressive symptoms (yes or no). Information on depressive symptoms was acquired by the question, "Over the past 12 months, have you had any depressive symptoms?"

#### Occupational characteristics

The occupational characteristics included job type (white collar, service, or blue collar, based on the Korean Standard Classification of Occupations), employment status (temporary or regular), shift work (yes or no) and working time (<40 hours a week, 40–48 hours a week, 49–60 hours a week, or ≥61 hours a week).

#### Independent variables

##### Physical, chemical, and biological risk factors

The physical risk factors consisted of exposure to vibration, noise, high temperatures, and low temperatures. Chemical risk factors comprised exposure to mist, dust, or fumes, organic solvents, and chemical agents. Biological risk factors consisted of exposure to infectious agents. In the questionnaire, the components of risk factors were defined as "vibrations from hand tools and machinery", "noise so loud that workers had to raise their voice to talk to people", "high temperatures that make workers perspire even when not working", "low temperatures, regardless of indoor or outdoor", "breathing in mist, dust (wood or mineral) or fumes (from welding or exhaust gas)", "breathing in vapors such as organic solvents and thinners", "handling or being in direct contact with infectious agents (waste, body fluids, experimental materials)". The participants answered questions about each component of the risk factors according to a seven-point scale (all of the working time, almost all of the working time, 3/4 of the working time, half of the working time, 1/4 of the working time, almost never, never). These responses were divided into exposure (all of the working time, almost all of the working time, 3/4 of the working time, half of the working time, 1/4 of the working time) and lack of exposure (almost never, never). Next, the physical risk exposure group was defined as participants who reported being exposed to one or more risk factors among vibration, noise, high temperatures, and low temperatures. The chemical risk exposure group and biological risk exposure group were defined in the same way.

##### Psychosocial risk factors

The psychosocial risk factors were divided into five categories (job demand, job control, social support, job insecurity, lack of reward). Each category was evaluated by a combination of various questions used in the Kim’s 2011 study "Secondary analysis of KWCS: Causes of absenteeism due to disease in employed women” [37]. Job demand was evaluated by seven questions: working at very high speed, working to tight deadlines, suspension of working due to unforeseen problems, strict quality standards, self-assessment of working, solving unforeseen problems on your own, and insufficient working time. Job control was evaluated by seven questions: the possibility of spending time on personal problems during work, difficulty changing the order of your tasks/methods of work/speed or rate of work, freedom to express an opinion about choosing a co-worker, ability to take a break when you wish, and freedom to express an opinion about important decisions. Social support was evaluated by eight questions: support by co-workers, support by managers, existence of friends in the workplace, work feedback from managers, being treated with personal respect, resolving conflicts, organization of tasks or ordering tasks, participation in the decision making process. Job insecurity was evaluated by two questions: possibility of loss of the job in the next 6 months, and possibility of getting a similar job upon quitting your job. Finally, lack of reward was evaluated by one question: satisfaction with current earnings. Each question was assigned one point. Using the median as a threshold, each category was divided into low and high groups. The detailed classification was as follows: job demand (3 ≥ as low, 4 ≤ as high), job control (3 ≥ as low, 4 ≤ as high), social support (1 ≥ as low, 2 ≤ as high), job insecurity (0 as low, 1 as high), and lack of reward (0 as low, 1 as high). Cronbach’s alpha values are 0.639 for job demand, 0.680 for job control, and 0.775 for social support. Because job insecurity and lack of reward consisted of one or two questions, Cronbach’s alpha values for job insecurity and lack of reward were not calculated.

##### The dependent variable

The dependent variable was identified using the question "Over the past 12 months, have you had sleep disturbance symptoms?". If a participant responded "yes", we considered the worker to be suffering from sleep disturbance.

### Statistical methods

A descriptive analysis was carried out on general and occupational characteristics. We used simple logistic regression analysis to determine the crude odds ratios between workplace risk factor exposure and sleep disturbance. In addition, adjusted odds ratios were calculated with adjustment of general and occupational characteristics using multivariate logistic regression analysis. The level of statistical significance was 0.05.

## Results

### General and occupational characteristics of the study subjects

About 41.2% (2928 persons) of the respondents were female, and about 58.8% (4184 persons) were male. In the distribution by age, the majority were in their 30s, accounting for 28.9% (2,057) of all of the participants. Of the others, 1,880 (26.4%), 1,481 (20.8%), 1,155 (16.2%), and 539 (7.6%) were in their 40s, less than 30, 50s, and 60s or above. In the case of educational status, 3,172 participants (44.6%) had graduated from college or beyond. 2,966 participants (41.7%) had finished high school, and 973 participants (13.7%) had finished middle school or less. As for smoking habits, 3,884 (54.6%) were non-smokers, while 697 (9.8%) were past smokers, and 2,531 (35.6%) were current smokers. With regard to drinking frequency, 2,965 participants (41.7%) reported drinking once or less a week, 2,208 participants (31.1%) drank twice a week or more, and 1,938 participants (27.3%) were non-drinkers. Among all of the participants, 490 participants (6.9%) were diagnosed with hypertension, and the others (6,622, 93.1%) were not. 226 participants (3.2%) were diagnosed with obesity, and the others (6,885, 96.8%) were not. 113 participants (1.6%) reported having depressive symptoms, and the others (6,999, 98.4%) did not.

As for occupational characteristics, specifically, employment state, 4,568 participants (64.2%) were classified as regular workers and 2,544 participants (35.8%) were temporary workers. When classified by job type, 3,081 participants (43.3%) were white collar, 2,680 blue collar, and 1350 participants had a service job. As for the number of employees, the majority (3,295, 48.4%) worked in a workplace with 5–49 employees. 1,526 subjects (22.3%) were at jobs with less than 5 employees. 1,315 subjects (19.3%) worked at companies with 50–299 employees. 689 subjects (10.0%) worked at firms with more than 300 employees. With regard to tenure, 4,084 subjects (57.4%) reported 1–9 years on the job. 1,550 subjects (21.8%) had worked 10 years or more. 1,477 subjects (20.8%) had worked less than 1 year. As for time worked per week, half of the subjects (3,801, 53.5%) worked 40–48 hours a week. 1,624 subjects (22.8%) worked 49–60 hours a week. 1,105 subjects (15.5%) worked less than 40 hours a week. 581 subjects (8.2%) worked 61 or more hours a week. As for shift work, 6,337 subjects (89.1%) were non-shift workers and 774 subjects (10.9%) were shift workers (Table 1).

**Table 1:** Number of workers with sleep disturbance by general and occupational characteristics

			**Sleep disturbance**				
	**N**	**%**	**No**	**(%)**	**Yes**	**(%)**	p-value*
Total	7112	100.0	6876	96.7	236	3.3	
Gender							
Female	2928	41.2	2833	96.8	95	3.2	0.771
Male	4184	58.8	4043	96.6	141	3.4	
Age							
<30	1481	20.8	1442	97.4	39	2.6	0.524
30-39	2057	28.9	1985	96.5	72	3.5	
40-49	1880	26.4	1812	96.4	68	3.6	
50-59	1155	16.2	1118	96.8	37	3.2	
≥60	539	7.6	519	96.3	20	3.7	
Educational status							
Middle school or below	973	13.7	936	96.2	37	3.8	0.047
High school	2966	41.7	2886	97.3	80	2.7	
College or above	3172	44.6	3053	96.2	119	3.8	
Smoking							
Non-smoker	3884	54.6	3751	96.6	133	3.4	0.847
Ex-smoker	697	9.8	674	96.7	23	3.3	
Smoker	2531	35.6	2451	96.8	80	3.2	
Drinking frequency							
None	1938	27.3	1891	97.6	47	2.4	0.026
≤1 per week	2965	41.7	2862	96.5	103	3.5	
≥2 per week	2208	31.1	2122	96.1	86	3.9	
Employment status							
Regular	4568	64.2	4430	97.0	135	3.0	0.061
Temporary	2544	35.8	2446	96.1	98	3.9	
Job type							
White collar	3081	43.3	2966	96.3	115	3.7	0.006
Service	1350	19.0	1324	98.1	26	1.9	
Blue collar	2680	37.7	2586	96.5	94	3.5	
Number of employees							
<5	1526	22.3	1474	96.6	52	3.4	<0.001
5-49	3295	48.4	3210	97.4	85	2.6	
50-299	1315	19.3	1246	94.8	69	5.2	
≥300	689	10.0	667	96.8	22	3.2	
Tenure (yr)							
<1	1477	20.8	1431	96.9	46	3.1	0.764
1-9	4084	57.4	3943	96.5	141	3.5	
≥10	1550	21.8	1501	96.8	49	3.2	
Working time (hr/wk)							
<40	1105	15.5	1083	98.0	22	2.0	<0.001
40-48	3801	53.5	3697	97.3	104	2.7	
49-60	1624	22.8	1562	96.2	62	3.8	
≥61	581	8.2	533	91.7	48	8.3	
Shift work							
No	6337	89.1	6153	97.1	184	2.9	<0.001
Yes	774	10.9	722	93.3	52	6.7	
Hypertension							
No	6622	93.1	6412	96.8	210	3.2	0.011
Yes	490	6.9	464	94.7	26	5.3	
Obesity							
No	6885	96.8	6667	96.8	218	3.2	<0.001
Yes	226	3.2	209	92.5	17	7.5	
Depressive symptoms							
No	6999	98.4	6811	97.3	188	2.7	<0.001
Yes	113	1.6	65	57.5	48	42.5	

*based on a chi-squared test.

### Association between characteristics and sleep disturbance

Of all of the participants, 236 subjects (3.3%) reported having a sleep disturbance. In the gender distribution, 95 subjects (3.2%) were female, and the others (141, 3.4%) were male. There were no significant differences in the gender or age of those with sleep disturbance. Participants with a college education or beyond (119, 3.8%) or middle school graduation or below (37, 3.8%) had significantly more sleep disturbance than high school graduates (80, 2.7%). Furthermore, the sleep disturbance rate in the group drinking alcohol twice a week or more (86, 3.9%) was significantly higher than that in the group drinking once a week or less. There was no difference in sleep disturbance by smoking habits. There was a significantly higher sleep disturbance rate in the group that had depressive symptoms than in the group that did not have such symptoms. In case of hypertension and obesity, the groups that had hypertension or obesity had a significantly higher sleep disturbance rate than the groups that did not have hypertension or obesity.

Among the occupational characteristics, white collar workers had a statistically significantly greater sleep disturbance rate than service workers or blue collar workers. The subjects who worked at workplaces with 50–299 employees had a significantly higher sleep disturbance rate than the other groups. The subjects who worked more than 61 hours a week also had a significantly higher sleep disturbance rate than the other groups. Compared with the non-shift worker group, the shift worker group had significantly greater sleep disturbance. In the categories of employment status and tenure, there was no significant difference in the sleep disturbance rate.

### Logistic regression analysis of various risk factors and sleep disturbance

To evaluate the association between physical, chemical, biological, and psychosocial risk factors and sleep disturbance, a chi-squared test was run. Each group that was exposed to physical, chemical, or biological risk factors was significantly associated with sleep disturbance. More specifically, exposure to noise, high temperatures, and low temperatures among the physical risk factors and mist (dust, fumes), and organic solvents and chemical agents among the chemical risk factors were significantly associated with sleep disturbance. A higher proportion of the group that was exposed to vibration had sleep disturbance than the group that was not exposed to vibration; however, there was no statistically significant difference. Among psychosocial risk factors, high job demand, inadequate social support and lack of reward were significantly associated with sleep disturbance (Table 2).

**Table 2:** Relationship between various risk exposures and sleep disturbance

			**Sleep disturbance**				
	**N**	**%**	**No**	**%**	**Yes**	**%**	p-value*
**Physical risk factors**							
Unexposed	4348	61.1	4234	97.4	114	2.6	<0.001
Exposed	2763	38.9	2642	95.6	121	4.4	
Vibration							
Unexposed	5324	74.9	5158	96.9	166	3.1	0.127
Exposed	1786	25.1	1717	96.1	69	3.9	
Noise							
Unexposed	5288	74.4	5131	97.0	157	3.0	0.005
Exposed	1823	25.6	1744	95.7	79	4.3	
High temperature							
Unexposed	5671	79.8	5520	97.3	151	2.7	<0.001
Exposed	1441	20.2	1356	94.1	85	5.9	
Low temperature							
Unexposed	6356	89.4	6164	97.0	192	3.0	<0.001
Exposed	755	10.6	711	94.2	44	5.8	
**Chemical risk factors**							
Unexposed	5484	77.1	5322	97.0	162	3.0	0.002
Exposed	1627	22.9	1553	95.5	74	4.5	
Mist, dust, or fumes							
Unexposed	5756	81.0	5579	96.9	177	3.1	0.025
Exposed	1354	19.0	1296	95.7	58	4.3	
Organic solvents							
Unexposed	6645	93.4	6437	96.9	208	3.1	0.001
Exposed	466	6.6	438	94.0	28	6.0	
Chemical agents							
Unexposed	6612	93.0	6406	96.9	206	3.1	0.001
Exposed	500	7.0	470	94.0	30	6.0	
**Biological risk factors**							
Infectious agents							
Unexposed	6803	95.7	6590	96.9	213	3.1	<0.001
Exposed	308	4.3	285	92.5	23	7.5	
**Psychosocial risk factors**							
High job demand							
Low	5332	75.0	5224	98.0	108	2.0	<0.001
High	1779	25.0	1651	92.8	128	7.2	
Insufficient job control							
Low	4118	57.9	3972	96.5	146	3.5	0.211
High	2993	42.1	2903	97.0	90	3.0	
Inadequate social support							
Low	3752	52.7	3650	97.3	102	2.7	0.003
High	3360	47.3	3226	96.0	134	4.0	
Job insecurity							
Low	2963	41.8	2871	96.9	92	3.1	0.435
High	4127	58.2	3985	96.6	142	3.4	
Lack of reward							
Low	4265	60.1	4158	97.5	107	2.5	<0.001
High	2831	39.9	2702	95.4	129	4.6	

*based on a chi-squared test.

In univariate logistic regression analyses, exposure to any of the risk factors was significantly associated with sleep disturbance. The odds ratio (OR) and 95% confidence interval (CI) of each risk factor was as follows: physical risk factor exposure group (OR 1.70, 95% CI 1.31-2.21), chemical risk factor exposure group (OR 1.57, 95% CI 1.18-2.07), and biological risk factor exposure group (OR 2.48, 95% CI 1.59-3.88). Psychosocial risk factors that were significantly associated with sleep disturbance were high job demand (OR 3.74, 95% CI 2.88-4.86), inadequate social support (OR 1.50, 95% CI 1.15-1.95), and lack of reward (OR 1.86, 95% CI 1.43-2.41) (model I). Covariates that were significantly associated with sleep disturbance among general characteristics were educational status, drinking frequency, hypertension, obesity, and depressive symptoms. After controlling for these covariates, including age and gender, multivariate logistic regression analyses were conducted. The odds ratio was 1.31 (95% CI 0.94-1.83) for physical risk factor exposure, 1.00 (95% CI 0.69-1.45) for chemical risk factor exposure, and 1.67 (95% CI 0.99-2.80) for biological risk factor exposure. Although these risk factors were not significantly associated with sleep disturbance, high job demand (OR 3.12, 95% CI 2.34-4.17), inadequate social support (OR 1.52, 95% CI 1.13-2.05), and lack of reward (OR 1.46, 95% CI 1.09-1.95) were still significantly associated with sleep disturbance (Model II). After additional adjustments for occupational characteristics such as job type, number of employees, weekly working hours, and shift work; physical risk factor exposure (OR 1.47, 95% CI 1.04-2.07), high job demand (OR 2.93, 95% CI 2.16-3.98), inadequate social support (OR 1.57, 95% CI 1.14-2.15), and lack of reward (OR 1.45, 95% CI 1.08-1.96) were significantly associated with sleep disturbance in a multivariate logistic regression analysis. Chemical risk factor exposure (OR 1.13, 95% CI 0.77-1.64) and biological risk factor exposure (OR 1.26 95% CI 0.72-2.21) were not significant, as in model II (Model III) (Table 3). To determine the multicollinearity between variables that were used in this study, the variance inflation factor (VIF) value was calculated. The VIF value of most of the variables was nearly 1. The highest VIF value was 2.08 for job type, so we concluded that there is almost never multicollinearity between variables.

**Table 3:** Odds ratios of variables associated with sleep disturbance

	Model I*		**Model II†**		**Model III‡**	
	**OR**	**95% CI**	**OR**	**95% CI**	**OR**	**95% CI**
Gender (reference [ref]: female)			0.85	0.61 - 1.17	0.77	0.54 - 1.09
Age			1.01	0.99 - 1.02	1.01	0.99 - 1.02
Educational status(ref: Middle school or below)						
High school			0.73	0.46 - 1.16	0.69	0.42 - 1.12
College or above			1.17	0.71 - 1.92	0.93	0.51 - 1.69
Drinking frequency (ref: none)						
≤1 per week			1.32	0.90 - 1.95	1.26	0.85 - 1.88
≥2 per week			1.72	1.14 - 2.61	1.78	1.17 - 2.72
Hypertension (ref: no)			1.28	0.78 - 2.11	1.10	0.65 - 1.85
Obesity (ref: no)			1.60	0.89 - 2.89	1.73	0.93 - 3.21
Depressive symptoms (ref: no)			17.36	11.12 - 27.09	15.16	9.49 - 24.23
Job type (ref: white collar)						
Service					0.45	0.26 - 0.76
Blue collar					0.53	0.33 - 0.84
Working time/wk (ref: 40–48 hr)						
<40					1.21	0.72 - 2.02
49-60					1.31	0.91 - 1.88
≥61					2.32	1.51 - 3.57
Shift work (ref: no)					2.49	1.68 - 3.70
Number of employees (ref: <5)						
5-49					0.62	0.42 - 0.91
50-299					1.17	0.76 - 1.81
≥300					0.59	0.33 - 1.05
Physical risk exposure (ref: unexposed)	1.70	1.31 - 2.21	1.31	0.94 - 1.83	1.47	1.05 - 2.07
Chemical risk exposure (ref: unexposed)	1.57	1.18 - 2.07	1.00	0.69 - 1.45	1.13	0.77 - 1.64
Biological risk exposure (ref: unexposed)	2.48	1.59 - 3.88	1.67	0.99 - 2.80	1.26	0.72 - 2.21
High job demand (high/low)	3.74	2.88 - 4.86	3.12	2.34 - 4.17	2.93	2.16 - 3.98
Insufficient job control (high/low)	0.84	0.64 - 1.10	0.76	0.57 - 1.02	0.78	0.57 - 1.05
Inadequate social support (high/low)	1.50	1.15 - 1.95	1.52	1.13 - 2.05	1.57	1.14 - 2.15
Job insecurity (high/low)	1.11	0.85 - 1.45	1.04	0.78 - 1.39	1.08	0.80 - 1.46
Lack of reward (high/low)	1.86	1.43 - 2.41	1.46	1.09 - 1.95	1.45	1.08 - 1.96

* Model I: odds ratio by independent univariate logistic regression analysis.

†Model II: multivariate logistic regression analysis, adjusted for general characteristics simultaneously.

‡Model III: multivariate logistic regression analysis, adjusted for general and occupational characteristics simultaneously.

## Discussion

This study was conducted to evaluate the association between physical, chemical, biological, and psychosocial risk factor exposure and sleep disturbance in a nationwide representative sample of paid workers. It was found that groups that were exposed to workplace risk factors tended to have higher odds ratios of sleep disturbance compared with groups that did not. We also expected that physical, chemical, biological, and psychosocial risk factors would be related to occupational variables, such as long work hours, shift work, and job type. Although not shown in the tables, it was found that the proportions of blue collar workers, of those who worked more than 60 hours a week, and of shift workers were significantly higher in the physical risk factor exposure group. The proportions of blue collar workers and of workers that worked more than 60 hours a week was significantly higher in the chemical risk factor exposure group. In the biological risk factor exposure group, the proportions of blue collar and shift workers was significantly higher. In addition, in the subcategories of the psychosocial risk factor exposure group, the proportion of blue collar workers, workers with long hours, and shift workers was significantly higher. After adjusting for these variables, the physical and psychosocial risk factors retained a statistically significant relationship with sleep disturbance, so it could be concluded that these workplace risk factors contributed to the sleep disturbance of the workers.

Although it could be difficult to compare the results of this study with those of other studies due to different study methods, in previous studies, results similar to ours have been found. Azmoon et al. found that thermal comfort in the workplace was related to high quality sleep in a cross-sectional study [38]. Workers who have been exposed to organic solvents for a long period of time have a high prevalence of sleep apnea compared with the general population, and this can induce fatigue, daytime sleepiness, and decreased concentration [39,40]. However, the mechanism of the effect of the organic solvents on sleeping problems has not been clearly established. In another study on organic solvents, it was supposed that the association between organic solvents and sleeping problems was a result of selection bias [41]. Noise in the workplace is a strong stressor; it causes a diminution of quality of sleep through decreased sleeping time [42]. Heavy metals (mercury [43], lead [44], and manganese [45]) and chemical agents [46] cause sleep disturbance by affecting the nervous system and hormonal system. According to previous studies, vibration has an effect on the autonomic nervous system and induces sleep disturbances such as insomnia [35,47]. Among general and occupational characteristics, drinking frequency (more than twice a week), shift work, long working time (more than 61 hours a week), and depressive symptoms were found to be related to sleep disturbance in the present study. This result is consistent with previous studies [18,19,24-26]. Smoking, including passive smoking, has an arousal effect on the brain due to nicotine exposure and results in insufficient sleeping time by interrupting sleep initiation [48]. Besides, it is known that snoring and daytime sleepiness is caused by smoking [49]. However, Davila et al. reported that the association between smoking and sleep disturbance was not statistically significant [50]. In fact, the effect of smoking on sleep disturbance was not consistent in previous studies, and we could not find an association between smoking and sleep disturbance. Educational status was not statistically significant after adjusting confounding factors, both the high educational group (college graduation and above) and low educational group (middle school graduation and below) had a significantly higher prevalence of sleep disturbance in univariate analyses. The reason may be that workers who have attained an advanced education usually are at relatively higher status employment and higher status employees feel a greater obligation to work because, culturally, the concept of lifetime employment is stronger in Asia than in western countries. This leads to long working hours and insufficient sleeping time [22].

The mechanisms by which physical, chemical, and biological risk factors cause sleep disturbances have not been fully clarified. However, in general, increases in the hormone cortisol and the hypothalamic-pituitary-adrenal axis play an important role in sleep, and sleep disturbance can be induced by their impaired function [51]. Therefore, it can be supposed that physical, chemical, and biological risk factors of the workplace may directly affect the hypothalmic-pituitary-adrenal axis and increase cortisol, and consequently, sleep disturbance occurs. Second, hazardous chemicals can induce neurobehavioral symptoms including sleep disturbance by directly or indirectly affecting the neurotransmitters of the central and peripheral nervous system [52-54]. Third, psychological factors are an important cause of sleep disturbance. Therefore, concerns or anxiety about health status can be affected by direct exposure to handling hazardous materials, and increased stress by recognition of poor working conditions involving exposure to hazardous materials could induce a sleep disturbance [55]. Fourth, exposure to various risk factors can affect workers' overall health status including chronic diseases, and this may induce a sleep disturbance [56].

The findings of the present study on the association between psychosocial risk factors and sleep disturbance are almost the same as those of previous studies. According to a study using the Korean Occupational Stress Scale (KOSS) for 8,155 Korean workers, the group with high job stress had a higher odds ratio of insomnia than the group with low stress, and job stress was a cause of insomnia directly or indirectly affecting the hormonal system, lifestyle factors such as smoking and drinking, and health status such as depression [33]. In addition, in a Japanese cohort study, inadequate social support and lack of reward was significantly associated with perpetuation of insomnia, and overcommitment and high job strain were significantly associated with the occurrence of insomnia [57]. Murata et al. reported that high job demand was associated with difficulty of sleep maintenance and low quality of sleep, while low job control and inadequate social support were significantly associated with difficulty of falling asleep as well as difficulty maintaining sleep and low quality of sleep in a study of employees older than 35 years old [58]. Åkerstedt also reported that high job demands and inadequate social support were related to sleep disturbance, and the odds ratio for high job demand was relatively high (OR 2.15) compared with other factors in a cross-sectional study. Therefore, it was supposed that high job demand was a classical occupational stress factor [59]. In another study, interpersonal conflict and low job satisfaction were associated with an increased risk of insomnia, and this result is consistent with the present study [60]. The reasons that psychosocial stress can induce sleep disturbance or sleep problems like sleep apnea, are as follows. First, as mentioned earlier, impaired function of the hypothalamic-pituitary-adrenal axis can induce sleep disturbance by affecting the hormonal system [50]. Second, workers with sleep disturbance induced by chronic diseases, depression, or inappropriate lifestyle can experience an increase in job stress for other reasons like conflict with colleagues, impaired job performance, or employment instability. Third, confounding factors that were not adjusted for in the present study can exist, such as a lack of exercise or snoring [61].

This study has a number of limitations. Because this study used a sample of the second KWCS conducted by the Occupational Safety and Health Research Institute, given the cross-sectional nature of the study, an association between physical, chemical, biological, and psychosocial risk factors and sleep disturbance was found, but we cannot prove a causal relationship. Furthermore, the second KWCS was not a survey on sleep disturbance in the evaluation of methods of sleep disturbance, so it was evaluated by just one question about whether or not a sleep disturbance is present, not using a specialized evaluation tool such as the Pittsburgh Sleep Quality Index (PSQI) or a sleep disorders questionnaire. As a result, an overall evaluation of the quality or quantity of sleep was not achieved. In addition, information about sleep disturbance, obesity and hypertension was obtained by self-administered questionnaire; thus, there is a possibility of recall bias and decreased objectivity. Third, given the healthy worker effect, we could not exclude the possibility that workers with poor health status or sleep disturbance might have retired or left their job. Fourth, there is a deficiency in detailed workplace risk factor evaluations. We were not able to confirm the exposure root, exposure quantity, exposure duration, or whether or not protective equipment was being worn, and if these factors were confirmed, it is expected that an obvious association could be verified. Fifth, the prevalences of sleep disturbances and depressive symptoms were relatively lower than in other studies. As mentioned, this is because of the healthy worker effect and the fact that the present study did not use an objective evaluation tool. Also, the prevalence is variable according to the study population and disease criteria [62], and direct comparison is not appropriate between the present study that used one question for evaluation for sleep disturbance and depressive symptoms and other studies that used specialized questions for the evaluation of sleep disturbance and depressive symptoms. Sixth, for evaluation of psychosocial risk factors, combination of questions was used. However, the Cronbach’s alpha values were 0.639 for job demand, 0.680 for job control, and 0.775 for social support, so the reliability was not sufficiently high.

In spite of these limitations, the present study is meaningful in that this study is an epidemiological study that synthetically evaluated an association between physical, chemical, biological, and psychosocial risk factors of the workplace environment and sleep disturbance using a sample from the second KWCS, which is a nationwide representative survey of paid workers. In the results of the study, an association between various risk factors and sleep disturbance was found, not a causal relationship. In future studies, improving upon the limitations of this study, a causal relation could be confirmed by using a study design such as a cohort study that can clarify a sequential relationship, an objective sleep evaluation tool, and a specialized questionnaire for psychosocial risk factors, as well as gathering detailed information about workplace risk factors.

Sleep disturbance is a common disease, as mentioned in that introduction. If a worker has a problem sleeping, the productivity and profitability of the company will be decreased due to low work ability, absences, and accidents, and the worker’s quality of life will also be affected. Therefore, management of sleep disturbance is needed. In a study of Shahly et al., the average cost for accidents or errors due to insomnia was reported to be significantly higher than that due to other causes [63]. In America, 7.2% of costs due to accidents or errors in the workplace were found to be associated with insomnia [63], and if management for sleep disturbance is appropriate, productivity will be increased by decreasing the cost of accidents or errors. In Korea, the interest in sleep disturbance is relatively low, and studies on sleep disturbances are rare, compared with Europe, Japan, and the US. Therefore, in addition to the improvement of the workplace environment, such as lowering the intensity and duration of workers' exposure to physical, chemical, and biological risk factors and appropriate education about self-protection; management of psychosocial risk factors and interest in the sleep health of workers are needed. It is also necessary to identify other risk factors that could affect workers' sleep health and manage these factors to improve the sleep health of workers.

## Competing interests

The authors declare that they have no competing interests.

## Authors’ contributions

YSH, HCK, JHL and SGP designed the research. YSH, SHJ and BJL performed the statistical analysis and interpreted the data. YSH and HCK wrote the manuscript. SJC and KYR critically revised the manuscript. All authors read and approved the final manuscript.
